# Exploring data reduction strategies in the analysis of continuous pressure imaging technology

**DOI:** 10.1186/s12874-023-01875-y

**Published:** 2023-03-01

**Authors:** Mingkai Peng, Danielle A. Southern, Wrechelle Ocampo, Jaime Kaufman, David B. Hogan, John Conly, Barry W. Baylis, Henry T. Stelfox, Chester Ho, William A. Ghali

**Affiliations:** 1 Libin Cardiovascular Institute of Alberta, University of Calgary, Calgary, AB Canada; 2 O’Brien Institute for Public Health, University of Calgary, Calgary, AB Canada; 3 W21C Research and Innovation Centre, Cumming School of Medicine, GD01 Teaching Research & Wellness Building, University of Calgary, 3280 Hospital Drive, Calgary, NW Canada; 4 Department of Medicine, Cumming School of Medicine, University of Calgary, Calgary, AB Canada; 5 Department of Community Health Sciences, Cumming School of Medicine, University of Calgary, Calgary, AB Canada; 6 Infection Prevention and Control, Alberta Health Services, Calgary, AB Canada; 7 Snyder Institute for Chronic Diseases, Cumming School of Medicine, University of Calgary, Calgary, AB Canada; 8 Foothills Medical Centre, Special Services Building, Ground Floor, AGW5, Calgary, AB T2N 2T9 Canada; 9 Department of Critical Care Medicine, Cumming School of Medicine, University of Calgary, Calgary, AB Canada; 10 Alberta Health Services, Alberta, Canada; 11 Department of Medicine, Division of Physical Medicine & Rehabilitation, University of Alberta, Edmonton, AB Canada; 12 Division of General Internal Medicine, Cumming School of Medicine, University of Calgary, Calgary, AB Canada

**Keywords:** Data reduction, Big data, Data management, Continuous pressure imaging, Heat maps, Time series plots

## Abstract

**Background:**

Science is becoming increasingly data intensive as digital innovations bring new capacity for continuous data generation and storage. This progress also brings challenges, as many scientific initiatives are challenged by the shear volumes of data produced. Here we present a case study of a data intensive randomized clinical trial assessing the utility of continuous pressure imaging (CPI) for reducing pressure injuries.

**Objective:**

To explore an approach to reducing the amount of CPI data required for analyses to a manageable size without loss of critical information using a nested subset of pressure data.

**Methods:**

Data from four enrolled study participants excluded from the analytical phase of the study were used to develop an approach to data reduction. A two-step data strategy was used. First, raw data were sampled at different frequencies (5, 30, 60, 120, and 240 s) to identify optimal measurement frequency. Second, similarity between adjacent frames was evaluated using correlation coefficients to identify position changes of enrolled study participants. Data strategy performance was evaluated through visual inspection using heat maps and time series plots.

**Results:**

A sampling frequency of every 60 s provided reasonable representation of changes in interface pressure over time. This approach translated to using only 1.7% of the collected data in analyses. In the second step it was found that 160 frames within 24 h represented the pressure states of study participants. In total, only 480 frames from the 72 h of collected data would be needed for analyses without loss of information. Only ~ 0.2% of the raw data collected would be required for assessment of the primary trial outcome.

**Conclusions:**

Data reduction is an important component of big data analytics. Our two-step strategy markedly reduced the amount of data required for analyses without loss of information. This data reduction strategy, if validated, could be used in other CPI and other settings where large amounts of both temporal and spatial data must be analysed.

## Introduction

The development of big data and big data analytics in healthcare holds the promise of improving healthcare resource value, management practices, and patient outcomes, while decreasing healthcare costs[1, 2, 3, 4]. Human generated information is the most common source of big data based on a recent systematic review on healthcare analytics [77%][5]. In health care, large amounts of clinical data are being generated and collected on an unprecedented scale[6, 7]. With the wide adoption of electronic medical records, contact data on millions of patients are being collected and stored in an electronic format. New technologies can now collect a variety of health measures (e.g., blood pressure, oxygen saturation, glucose) on a continuous basis, generating large amounts of data[8]. This generation of large volumes of data poses challenges in data management and analysis[9, 10].

Big data analytics methodology is a multistep process that typically includes a concept statement or goal for the data mining application, dataset selection, pre-analysis data processing (transformation and/or reduction), application of one or more analytic approaches, evaluation and interpretation of results, and application and translation of findings[11, 12]. Data reduction is a necessary step in big data analysis. Approaches to this vary and include but are not limited to pure dimension reduction techniques, compression-based data reduction methods, and algorithms for summarization, de-duplication, and redundancy elimination. Selection of the approach depends on the nature of collected data as well as the computer resources. For example, dimension reduction can be applied to noisy data to identify and eliminate features that are unimportant. No prior assumption about the number of clusters is required. Compression-based data reduction methods are suitable in order to preserve the original data at the expense of resource consumption from additional computations and redundancy elimination methods will depend on the application models as to the selection of the method [13].

CPI technology monitors the interface pressure between a patient’s skin in contact with a support surface and provides assistance with patient repositioning to offload high or prolonged areas of pressure[14]. Prior to the trial, we have reported on a 9-patient pilot project with this technology demonstrating a statistically significant increase in patient turns or chair transfers to reduce interface pressures[15]. For this clinical trial we sought to determine whether the visual feedback provided by this technology can assist health care providers to diminish the likelihood of periods of excessive skin pressure and resultant pressure injuries. CPI data in addition to providing prompts to health care providers was also used as an outcome measure[16].

While CPI of hospitalized patients may be useful for both the prevention and management of these injuries, for each patient, every 72 h, 259,200 frames of pressure images at one-second intervals will be produced. This would create spatiotemporal data sets of approximately 6.7 GB on a single patient. As we anticipated enrolling 678 research subjects, our trial would collect in total an estimated 4.52 TB of data, creating challenges in data management and analysis not dissimilar to what other health researchers face in clinical trials of emerging health care technologies. The key pieces of information, collected in our trial, are pressure values and their duration on each body part while a patient laid on a hospital bed. The large amounts of data provided mean that it is necessary to find ways to summarize the data while avoiding loss of critical information.

Our group has conducted a clinical trial evaluating the effectiveness of continuous pressure imaging (CPI) technology on the reduction of pressure injuries [16]. In this paper we provide a case study demonstrating the potential utility of data reduction methods explored to pre-process CPI data from our trial with both spatial and temporal data points. The proposed technique helps us identify when a patient moved and how long patients stayed in the same position. This approach reduced the data size to a manageable level and allowed us to do summarization as needed. Our study is hypothesis generating and the approaches presented may be instructive to others. This big data case study and the data reduction strategies presented here are likely to be of value to readers and research teams facing similar big data challenges.

## Methods

### Data source

The objective of the randomized clinical trial was to test the efficacy of a CPI system in reducing both interface pressures and the risk of developing pressure injuries [16]. This trial used XSENSOR® Technology Corporation’s ForeSite PT™ Patient Turn System (referred to as the ForeSite PT™ system), which is composed of a repositioning reminder system utilizing CPI technology through a pressure-sensing mattress cover. The system consists of a flexible pressure sensing mattress covering that can collect up to 6136 pressure data points dispersed over a grid system of 52 rows by 118 columns, and an LCD monitor that displays the interface pressure readings in the form of a coloured pressure map with a timer and history bar for alerting and tracking patient repositioning. Health care professionals use the system to prompt repositioning of patients for pressure relief of target areas.

After informed consent was received in our study, a research nurse or assistant set up the ForeSite PT™ system on the patient’s hospital bed. The mattress cover was placed between the bed linen and mattress with the monitor mounted at the head of the bed. The ForeSite PT™ system was removed after 72 h of data collection or prior to this if the enrolled patient withdrew consent, died, was transferred to another unit/hospital, or was discharged from the hospital. Upon removal of the system, the interface pressure readings were downloaded in compressed Microsoft Office InfoPath 2003™ XML Form Template (XSN) formats. The files were then decompressed and converted to comma-separated values (CSV) files using the XSENSOR® Pressure Exposure Analyzer software. Detailed information about the clinical trial can be found in the published protocol [16] and on a trial registration website (ClinicalTrials.gov, NCT02325388).

To preserve the integrity of the clinical trial, for this paper we only utilized data from four study participants who did not complete the full 72 h and were not included in the primary trial analyses being done (i.e., analyses in which only individuals who completed the 72-h intervention are included). Patients who did not complete the 72-h intervention because either there was a planned transfer to another unit within 72 h of enrollment, the patient slept in a chair at night, medical status would have been negatively impacted if turned or repositioned or the patient received either palliative or end-of-life care (with death imminent). These data were used to explore how to pre-process pressure data before these analyses. The four patients provided CPI data ranging from 29 to 52 h in duration. The data size ranged from 2.7 to 4.9 GB for each study participant.

### Analytical plan

We designed a two-step strategy to reduce the data. First, we sampled the data at every 5 s (s), 30 s, 60 s, 120 s and 240 s to determine if we could reduce the frequency of pressure data examination without losing important information. Second, we used correlation coefficients to evaluate the similarity between adjacent frames to identify position changes and determine how we could coalesce frames collected during periods of stillness. Previous work on digital image correlation used similar methods and showed that correlation provides an effective measure on the similarity of images and enables us to identify the position and use of a single image to represent long static periods without position change [17]. Correlation coefficients of pressures between adjacent frames were calculated using pressure values from activated sensors. A sensor was activated if its pressure value was above 5 mmHg.

We defined two variables to illustrate the difference between adjacent frames. The first was the total number of sensors only activated in one of the two frames. The second variable was the sum of absolute pressure difference between two frames.

We divided a patient’s monitor time into three categories: time not-on-bed, active time on-bed with position changes within short time intervals, and on-bed stillness where there was no movement while on the bed. Not-on-bed time referred to the time period where the patient was not on the bed during the study period and defined as the sum of time periods when the number of activated sensors was less than 500, indicating that the patient was no longer lying or sitting on the bed. The use of less than 500 activated sensors to determine not on bed status was selected based on a visual inspection of all the individual images with less than 500 activated sensors. This excluded very few images while reducing the noise on the analysis. Identification of position changes depends on the selection of threshold of correlation coefficients. Active time was defined as the sum of all the time periods with continuous position changes. Two periods with position changes were combined if their time gap was less than 120 s to reduce the noise of calculation.

We used different plots to evaluate the performance characteristics and usefulness of different sampling frequencies and correlation coefficient thresholds to compress the data. Heat maps and scatter plots were used as a similarity measure to illustrate the spatial difference of pressure values between frames. Boxplots of differences in activated sensors and pressure differences were used to illustrate overall similarity of frames at different correlation coefficients. Lastly, a time series plot was used to show the continuous change of correlation coefficients, mean pressure values, number of activated sensor and number of sensors with pressure value > 40 mm Hg within 24 h for selected patients at different sampling frequency. The threshold value of > 40 mm Hg was based on the distribution of data from a pilot study we conducted [16] and the work of Agrawal et al. [18] that showed external pressures > 33 mm Hg would occlude blood vessel leading to underlying and surrounding tissues becoming anoxic. If the pressure continued at this level for a prolonged duration, cell death occurred resulting in soft tissue necrosis and eventual ulceration [18]. By studying the continuous change of correlation coefficients, we aim to provide justification of the face validity of the proposed technique and selection of appropriate thresholds for data reduction.

### Ethical considerations

Ethics approval for this study was obtained from the Conjoint Health Research Ethics Board of the University of Calgary (REB13-0794). Written informed consent was obtained from each participant after clarification of the study objectives and activities.

## Results

**Fig. 1:**
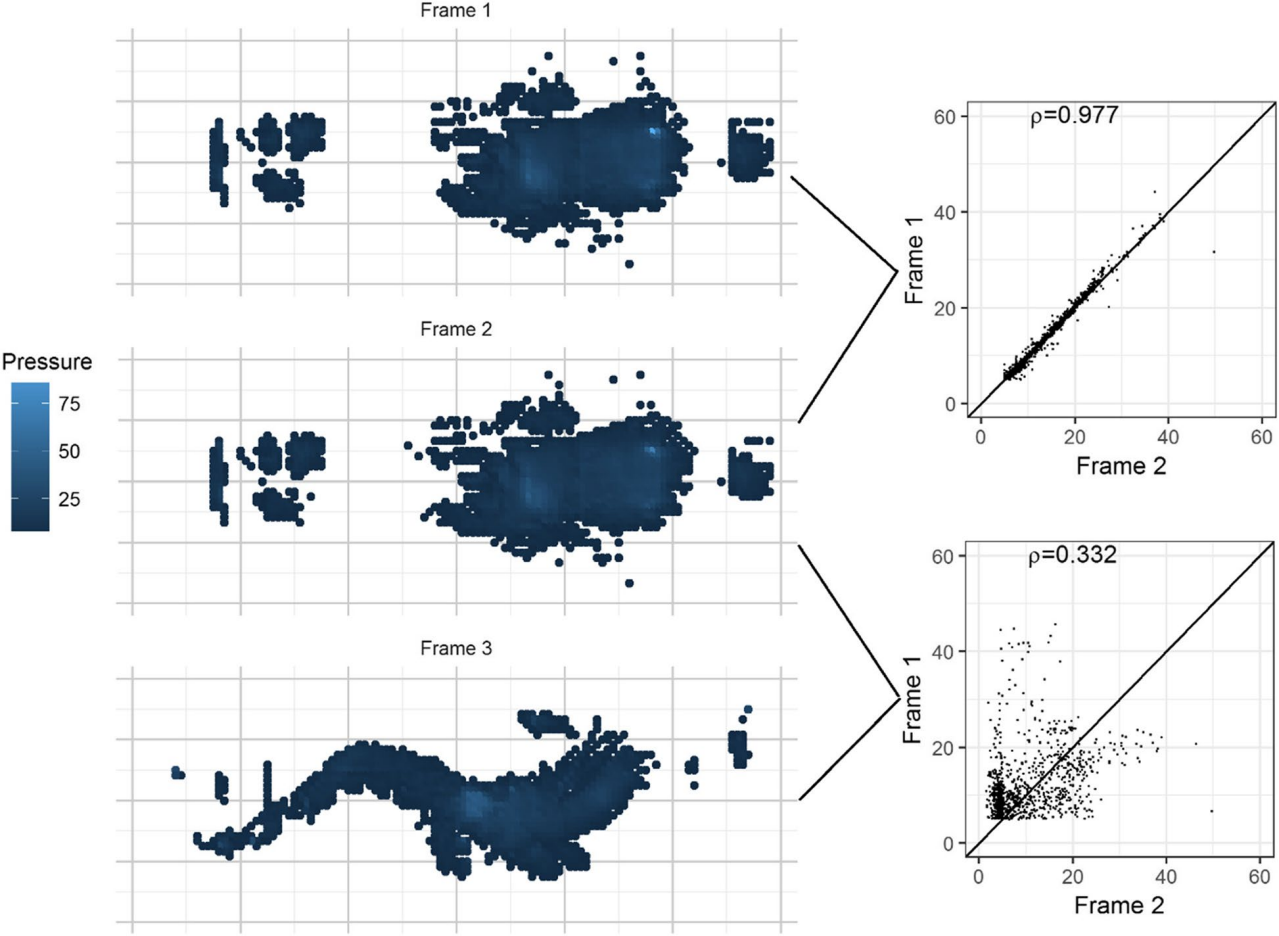
Heat map of pressure for a patient at three different changes and correlation of pressure between changes

As shown by the two heat maps (Fig. 1), Frames 1 and 2 were almost identical with negligible differences in pressure at the head position. Frames 1 and 2 had a correlation coefficient of 0.977. In contrast, the patient assumed a different lying position between frames 2 and 3. The correlation coefficient between frames 2 and frame 3 was very low (correlation coefficient = 0.332). If there is movement between two frames (e.g. the patient moves from one position to another position), the pressure value at the same position would change considerably, resulting in a lower correlation coefficient.

**Fig. 2:**
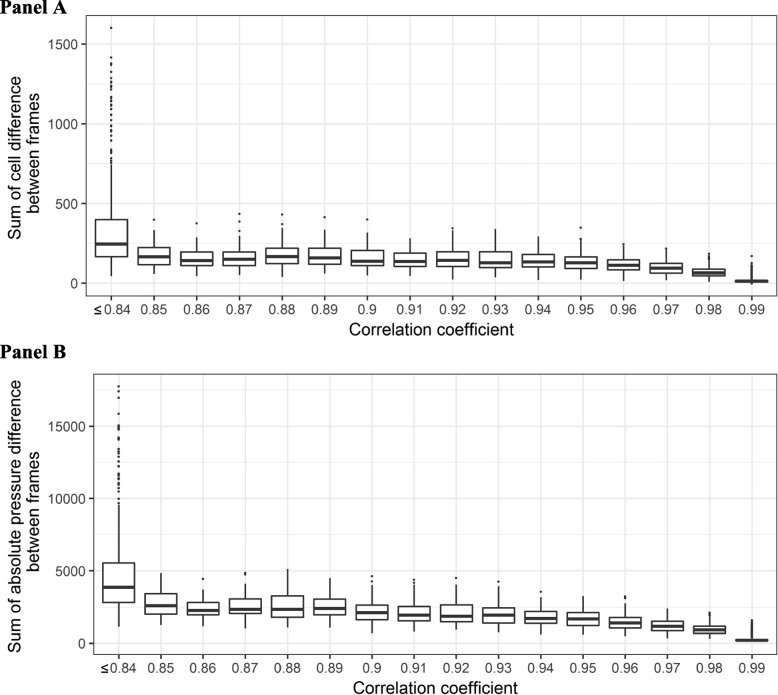
Boxplot of difference in activated sensors (Panel **A**) and absolute pressure difference (Panel **B**) between frames at different correlation coefficients

If the correlation coefficient was above 0.99, pairs of frames show almost no difference in the number of activated sensors (Fig. 2 Panel A) with absolute differences in pressure values close to zero (Fig. 2 Panel B). The difference in the number of activated sensors and pressure values increased when there was a decrease in the correlation coefficient.

**Fig. 3:**
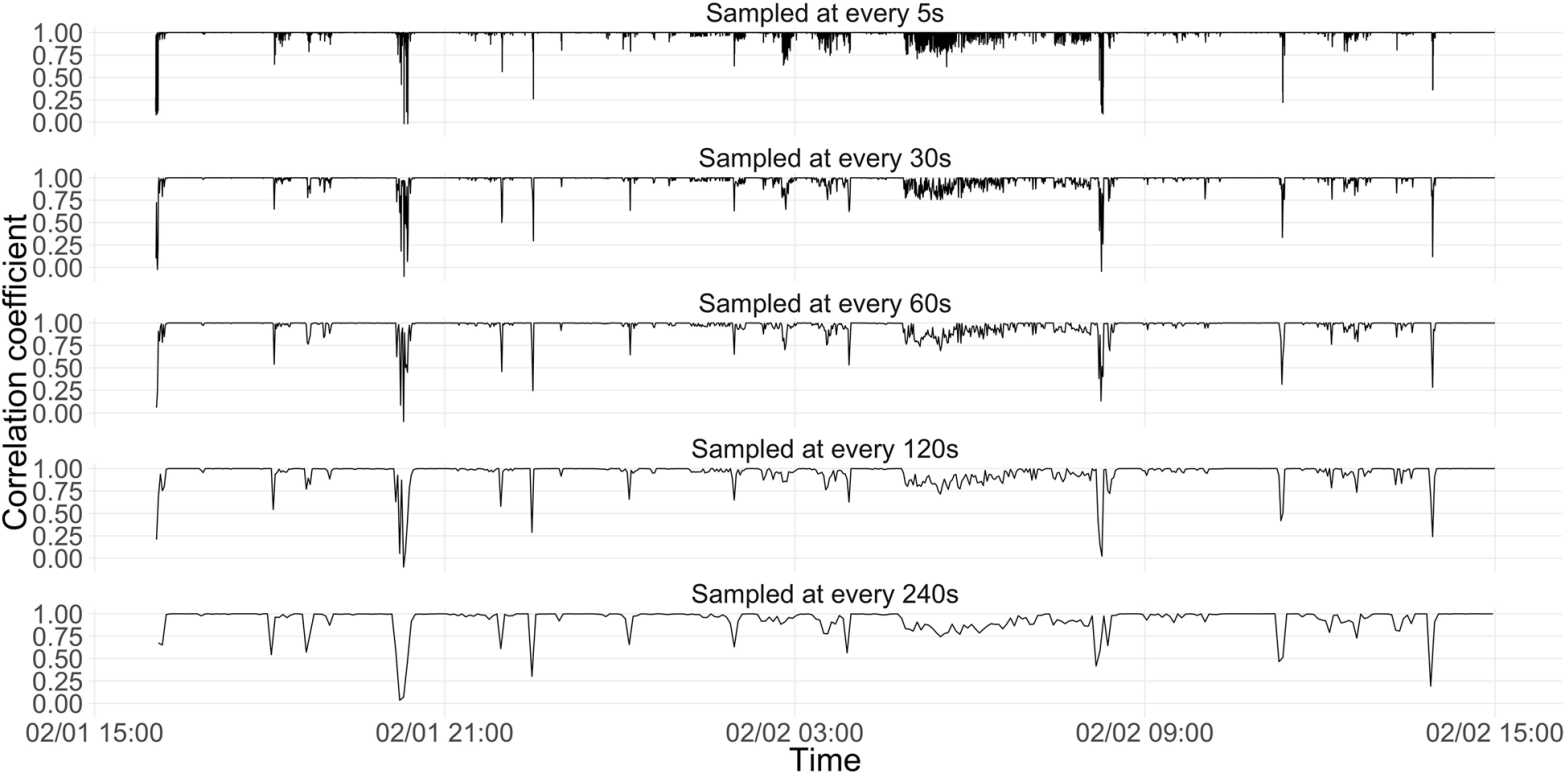
Change of correlation coefficient within 24 h at different sampling frequencies

Figure 3 plots correlation coefficients over 24 h in a selected patient at five sampling frequencies. The curve at low sampling frequencies (e.g. 120 s) was a similar but smoothed version of curves at higher sampling frequencies (e.g. 5 s) and having comparable times with correlation coefficient drops. Drops of correlation coefficient generated negative deflections on the curves, which indicated a complete position change as illustrated in Fig. 1. As shown in Fig. 3, the patient showed various position changes between periods of stillness. However, stillness sometimes was hard to define as patients may have only moved a limb without moving the trunk or torso of their body. As shown in the time period between 05:00 to 08:00, we observed continuous changes in the correlation coefficient with small deflections, which could represent a period of frequent patient repositioning.

**Fig. 4:**
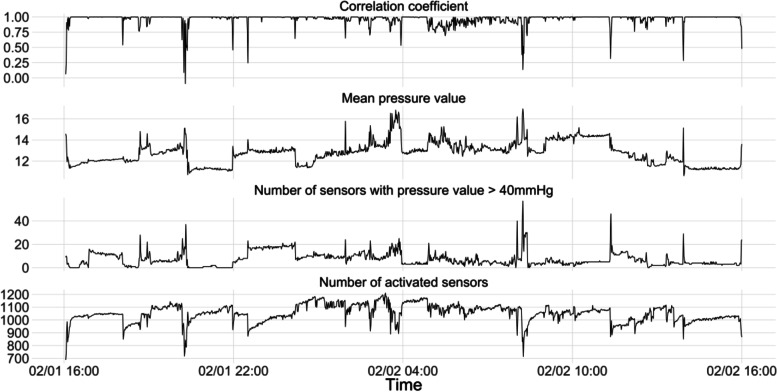
Change of correlation coefficients, mean pressure, and number of activated sensors (with pressure > 40 mmHg) over 24 h for a randomly selected patient at sampling frequency of every 60 s

Plot changes in correlation coefficients, mean pressure values, number of activated sensors, and number of sensors with pressure value > 40 mm Hg over 24 h at a sampling frequency of every 60 s are illustrated in Fig. 4. The peaks and flat sections from each curve aligned well with each other. A drop in correlation coefficient values corresponded to a sharp increase in mean pressure values, number of sensors with pressure values over 40 mm Hg, and a drop in the number of activated sensors. This aligns well with our perception of pressure changes during repositioning, which should be associated with decreases in contact areas and increases in mean pressure values.

**Fig. 5:**
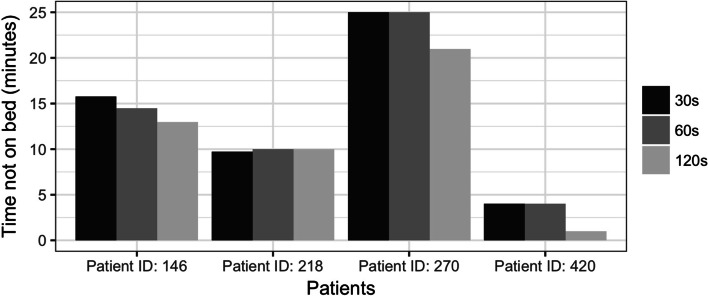
Time not-on-bed during study period for the four study participants

‘Not-on-bed’ times were similar at sampling frequencies of 30 s and 60 s for the four study participants (Fig. 5). In comparison, it appears that sampling at longer time intervals (i.e., every 120 s), resulting in substantially less data points, may be underestimating the ‘not-on-bed’ time.

**Fig. 6:**
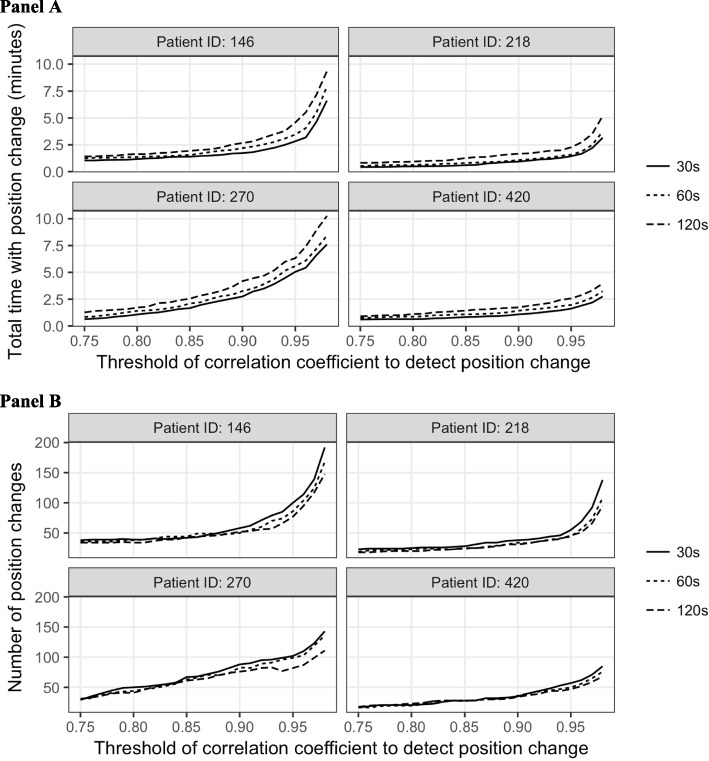
Sum of active time (movement) within 24 h from randomly selected four patients at different sampling frequency (every 30 s, 60 s, 120 s) (Panel **A**) and Total number of position changes in 24 h from four patients at different sampling frequency (every 30 s, 60 s, and 120 s) (Panel **B**)

Next, we defined a position change as occurring if correlation coefficients were below a pre-established threshold value. Use of high threshold value for the correlation coefficient could lead to the detection of partial position change, such as limb or head movement. As expected, active time depends on the selection of correlation coefficient thresholds (Fig. 6 Panel A). A low sampling frequency of 120 s slightly overestimated values. Higher thresholds for the correlation coefficient resulted in longer active time. A low sample frequency (e.g., 120 s) resulted in slightly longer active time than high sample frequencies (e.g., 60 s or 30 s). The differences of active time between different sampling frequencies decreased with a decrease in threshold values of correlation coefficients.

Lastly, the number of position changes captured decreased as we lowered the threshold of correlation coefficients (Fig. 6 Panel B). As expected, higher sampling frequencies identified more position changes. However, this difference was minimal once the threshold of correlation coefficient dropped below 0.9. If we set the threshold of correlation coefficient as 0.9, we observed around 20, 13, 50, and 25 active periods, respectively for the above four patients. The order of activity, based on the number of position changes, appeared consistent across all correlation coefficient values.

## Discussion

We utilized a two-step strategy to reduce raw spatial–temporal pressure data while preserving information. In the first step, a decrease of sampling frequency significantly reduced data size without compromising resolution. Based on our testing of the data, an optimal sampling frequency of every 60 s was found for the pressure data. The use of sampling frequency of every 60 s meant that we only needed to retain 1.7% (1/60) of raw data during pre-processing.

In the second step, we focused on the evaluation of the similarity between adjacent frames to identify lying position changes and coalesce the data during periods of stillness. Selection of an appropriate correlation coefficient threshold for position changes should strike a balance between major and minor position changes depending on the specific purpose of the analysis. The maximum number of active periods for 24 h is 80 if the threshold of correlation coefficient is set at 0.90. Therefore, we estimated that we would probably need ~ 160 frames within 24 h to represent the pressure states of a study participant. In total, this meant we needed ~ 480 frames (160*3), over 72 h, of collected data for our analyses without any relevant loss of information. These 480 frames represented ~ 0.185% of the raw data. The two-step process significantly reduced data size without the loss of information.

Data reduction is an important component of big data analytics. There are six core properties of big data—volume, variety, value, velocity, veracity, and variability[9]. Velocity refers to frequency of data streams into the data system and is one of the major challenges in our study. Data collection at an unnecessarily high frequency could overload the data system and/or lead to redundancy. The analyses and general approach presented here have helped us to reduce the collection frequency to make the data manageable while providing good representation of the pressure exposure of study participants. Data duplication is another issue for us. During periods of stillness, redundant information is collected without adding any new information about pressure distribution and lying positions of a patient. Similar to other data de-duplication schemes, we focused on the evaluation of the similarity of data. Correlation coefficients are a classic measure of similarity, and these have been applied to various applications, such as measuring reproducibility of RNA amplification reactions and separating noise and artifacts from ECG readings[19]. Our method focused on the reduction of data volume and velocity based on the characteristics of pressure imaging data.

To the best of our knowledge, our paper is unique in reporting on a data reduction approach for pre-processing of pressure data from a large clinical trial. There are other studies that have looked at interface pressure in a small group of participants over short or long periods of time. Peterson et al. looked at changes in interface pressures of routine repositioning in a convenience sample of 23 hospitalized bedridden patients[20]. They used a 24 × 24 in.^2^ sensory array pressure map with 2,304 sensors, and recorded interface pressure readings every 30 s for 4–6 h, which resulted in 15,784 pressure profiles. Their reason for capturing data every 30 s was not explained, but it did allow them to analyze pressure profiles of certain positions. Sakai et al. have used a sampling frequency of 60 s for pressure data in intensive care patients without providing justification[21]. In an article by Bogie et al., researchers developed an algorithm that used data mining for rapid information recovery and applied it to the analysis of interface pressure readings from 10 spinal cord injury patients to determine the effects of neuromuscular electrical stimulation on preventing pressure injuries[22]. A 40 × 38 cell sensor mat was used and 200 s of data was captured at a time. Participants were assessed 2–6 times with three 200 s readings per assessment, which resulted in 8,640,000 data points per subject. Although data reduction was not applicable in view of their small sample size and short periods of pressure readings, they did discuss how correlation coefficients could be used to align pressure readings at different times. They considered it to be not appropriate for their study because they were interested in detecting differences in pressure of the same locations over time. For our study, we noted differences in the capture of detail at 120 s and therefore did not test larger time frames.

### Limitations

Our study has an overriding limitation; the data that we analyze to showcase data reduction methodologies are unique to our study, and they are likely to differ from large complex databases that other researchers are using. This may limit the extent to which this methodologic data reduction case study is applicable to other big data scenarios. Nevertheless, we anticipate that some aspects of the methods presented can be applied to other data reduction challenges. There are some secondary study limitations relating to the pressure measurements that are central to our clinical trial. These include the challenges of data loss when patients are not in bed, unstable pressure measurements during position changes, and possibly some random ‘noise’ in the pressure data. We remind readers that this data reduction study is distinct from the data analysis for the clinical trial testing the efficacy of pressure sensing technology for pressure reduction and ulcer prevention.

There are also limitations relating to our use of the Pearson correlation coefficient. It assumes a linear relationship between variables and that the variables are normally distributed. Reassuringly, our sample size of pressure values was very large and near normally distributed with very few outlier measurements (see Fig. 1) – all features that make the Pearson correlation coefficient quite appropriate.

## Conclusion

Our study serves as a case study demonstrating a two-step strategy in data reduction of CPI data. The use of sample frequency at 60 s provided a reasonable representation of pressure exposure and would reduce the burden of data management. Correlation coefficients were an effective measure that allowed us to identify lying position changes of patients. Both of these strategies will allow us to more efficiently analyse the data to quantitatively understand how the use of CPI technology affects interface pressure, and to determine its effectiveness in reducing pressure-related injuries.

Data reduction methods are a necessary step in the era of adoption of new techniques that require big data analysis. Data reduction should be always considered in situations like ours, and ideally before starting data analysis. Our method may be generalizable to other data sets with spatiotemporal elements.

## Data Availability

The datasets used and/or analysed during the current study are available from the corresponding author on reasonable request.

## Abbreviations

CPI: Continuous pressure imaging
CSV: Comma-separated values
XSN: Microsoft Office InfoPath 2003™ XML Form Template
s: Seconds
